# Cognitive reflection vs. calculation in decision making

**DOI:** 10.3389/fpsyg.2015.00532

**Published:** 2015-05-07

**Authors:** Aleksandr Sinayev, Ellen Peters

**Affiliations:** Department of Psychology, The Ohio State UniversityColumbus, OH, USA

**Keywords:** numeracy, Cognitive Reflection Test, biases, financial outcomes, individual differences, dual-system theory

## Abstract

Scores on the three-item Cognitive Reflection Test (CRT) have been linked with dual-system theory and normative decision making (Frederick, 2005). In particular, the CRT is thought to measure monitoring of System 1 intuitions such that, if cognitive reflection is high enough, intuitive errors will be detected and the problem will be solved. However, CRT items also require numeric ability to be answered correctly and it is unclear how much numeric ability vs. cognitive reflection contributes to better decision making. In two studies, CRT responses were used to calculate Cognitive Reflection and numeric ability; a numeracy scale was also administered. Numeric ability, measured on the CRT or the numeracy scale, accounted for the CRT's ability to predict more normative decisions (a subscale of decision-making competence, incentivized measures of impatient and risk-averse choice, and self-reported financial outcomes); Cognitive Reflection contributed no independent predictive power. Results were similar whether the two abilities were modeled (Study 1) or calculated using proportions (Studies 1 and 2). These findings demonstrate numeric ability as a robust predictor of superior decision making across multiple tasks and outcomes. They also indicate that correlations of decision performance with the CRT are insufficient evidence to implicate overriding intuitions in the decision-making biases and outcomes we examined. Numeric ability appears to be the key mechanism instead.

## Introduction

Scores on the three-item Cognitive Reflection Test (CRT) have been linked with dual-system theory and normative decision-making patterns (Frederick, 2005). In particular, the CRT is thought to measure monitoring of System 1 intuitions such that, if cognitive reflection is high enough, intuitive errors will be detected and the problem will be solved. However, CRT items also require numeric ability to be answered correctly. In two studies, we examined whether the CRT was predictive of superior decision making because it measures the ability to check intuitions and/or the ability to solve numeric calculations.

## The cognitive reflection hypothesis

The CRT is a popular three-item test (Frederick, 2005) thought to assess cognitive reflection because the items bring to mind intuitive but wrong solutions that have to be overridden. The prototypical CRT problem is the bat and ball problem: “A bat and a ball cost $1.10. The bat costs $1.00 more than the ball. How much does the ball cost?” The response “10 cents” is thought to come to mind for most, if not all, people, and many people answer “10 cents.” Some people realize that the intuitive response is incorrect, however, and researchers have believed that calculating the correct answer is straightforward at that point: “catching [the] error is tantamount to solving the problem” (Frederick, 2005, p. 27). Kahneman (2011) called the bat and ball problem “a test of people's tendency to answer questions with the first idea that comes to mind, without checking it” (p. 65). Consistent with this view, we define Cognitive Reflection as the tendency to check and detect intuitive errors, and call the hypothesis that it is the important aspect of the CRT, the Cognitive Reflection Hypothesis.

In support of the Cognitive Reflection Hypothesis, Frederick (2005) briefly noted several pieces of unpublished evidence. In particular, people who responded correctly sometimes wrote the intuitive answer in the margin and described thinking about the intuitive answer in verbal reports, indicating that the intuition did come to mind. People who answered incorrectly thought the bat and ball problem was easier than those who answered correctly (incorrect responders judged the proportion of others who answered correctly to be higher than correct responders did), indicating that those who responded intuitively were unaware that the intuition was wrong. Inconsistent with this reasoning, however, De Neys et al. (2013) found that correct responders were more confident about their responses than incorrect responders. Frederick (2005) also noted that some people who perform badly on the CRT nonetheless are able to solve similar problems that do not have incorrect intuitive solutions (e.g., “a banana and a bagel cost 37 cents. The banana costs 13 cents more than the bagel”). However, Bourgeois-Gironde and Van der Henst (2009) subsequently demonstrated that most people answer these problems incorrectly anyway (58% incorrect; see also Mastrogiorgio and Petracca, 2014).

Alter et al. (2007) provided evidence consistent with the CRT assessing an increased tendency to check intuitions. In particular, they found that participants who read the CRT in a degraded font (which presumably increased information processing) answered correctly more often than participants who read it in a normal font. However, the effect was not limited to tasks that require checking and inhibiting intuitive responses. Diemand-Yauman et al. (2011) demonstrated that disfluent fonts improved performance on a wide range of tasks (including ones with and without intuitive responses). Their results indicate that the improvements in CRT performance may have been due to some other process such as a more general increase in deliberation rather than a specific increase in intuition checking. In sum, although some evidence exists that the CRT measures cognitive reflection, the same evidence is also consistent with it measuring other constructs.

## Dual-systems explanation

To explain his findings, Frederick (2005) invoked a dual-systems model of decision making. In it, intuitive System 1 processes are quick and effortless, whereas deliberative System 2 processes are slow and controlled. System 1 quickly makes an intuitive response available in decision making; System 2 then may check the response and engage in further reasoning if an error is detected (Kahneman and Frederick, 2002; Kahneman, 2003). Importantly, System 2 is activated only after System 1 processing is complete. This temporal rigidness distinguishes it from dual-process explanations of judgment and decision making that posit more interdependencies between the two modes of thought (Loewenstein et al., 2001; Slovic et al., 2004). Many biases are said to occur due to System 1's incorrect intuitions, so that people who check their intuitions (e.g., those scoring high on the CRT) should be less biased decision makers.

Consistent with this prediction, several studies have found correlations between the CRT and decision biases. In his original paper, Frederick (2005) found that people with lower CRT scores tended to be more impatient and risk averse, therefore failing to maximize expected utility. Oechssler et al. (2009) also found that people higher on the CRT were less likely to commit conjunction fallacies and conservatism in probability updating. Other researchers have found expected CRT correlations with probability updating, base rate neglect, and under/over confidence (Hoppe and Kusterer, 2011), regression to the mean, Bayesian reasoning errors, and framing effects (Toplak et al., 2011), performance on Wason selection and denominator neglect tasks (Toplak et al., 2014), and moral judgments (Paxton et al., 2012; Royzman et al., 2014). That CRT scores correlate with fewer judgment and decision biases has been interpreted as indicative of bias avoidance requiring one to check and correct intuitions and, therefore, as support for a dual-systems explanation of decision making (Thaler and Sunstein, 2008; Kahneman, 2011).

Each of these researchers assumes that differences in CRT performance indicated differences in the ability to detect and correct incorrect intuitions (i.e., the Cognitive Reflection Hypothesis). They also implicitly assume that numeric ability is an irrelevant detail when it comes to solving CRT and related problems. Contrary to this view, however, Baron et al. (2014) recently found that traditional CRT problems have no more predictive power with respect to moral preferences than similar arithmetic items without intuitive answers. These findings suggest that numeric ability may be important to CRT performance.

## The numeracy hypothesis

Other researchers include CRT items in measures of numeric ability, implying that the CRT is not substantially different from other math tests (Weller et al., 2013). In fact, four of the five published studies employing exploratory or confirmatory factor analyses concluded that CRT and other numeracy items load on the same factor (Weller et al., 2013; Baron et al., 2014; Låg et al., 2014; Study 1 of Liberali et al., 2012; see their Study 2 for the one exception). Baron et al. (2014) furthermore, concluded that CRT items were more similar to math items without intuitive answers than they were to non-numeric verbal problems that had CRT-like intuitive answers.

Numeric ability itself has been associated with superior performance in a variety of judgment and decision tasks, making it plausible that numeracy may account for at least part of the CRT's association with better decision making. For example, Peters et al. (2006) found lower numeracy was related to more framing and format effects as well as denominator neglect. More numerate individuals, on the other hand, were less influenced by non-numerical information such as mood states and they demonstrated greater number-related affective reactions and sensitivity to different levels of numeric risk (Peters et al., 2009; see Reyna et al., 2009; Peters, 2012; for reviews). Numeracy effects are not limited to lab studies. McArdle et al. (2009) demonstrated that the more numerate accrue more wealth (even after accounting for demographic characteristics and other cognitive abilities, for example, working and long term memory), perhaps because the more numerate are less risk averse in their investments. We call the view that the CRT is primarily a measure of numeric ability and that numeric ability drives the CRT's ability to predict better decisions, the Numeracy Hypothesis.

## Modeling cognitive reflection and numeric ability

Researchers have begun to recognize that the processes underlying CRT performance may include both cognitive reflection and numeric ability (Böckenholt, 2012b; Del Missier et al., 2012; Campitelli and Gerrans, 2014). Böckenholt (2012b) and Campitelli and Gerrans (2014), for example, assumed that solving a CRT problem required all participants initially to think of the incorrect intuitive response; then, their individual responses were determined in a two-step process of cognitive reflection and (if cognitive reflection was high enough to detect the intuitive error) numeric ability. For example, the bat and ball problem brings to mind an intuitive response (10 cents). If cognitive reflection is high enough, a person checks the response and determines it is wrong ($1.10 + $0.10 ≠ $1.10) and proceeds to the next step. To answer correctly (5 cents), the person must have the knowledge to set up the appropriate equation ($1.00 + *x* + *x* = $ 1.10); they must also have the capacity to solve the equation. If numeric ability is not high enough, an idiosyncratic non-intuitive error will emerge. In other CRT items, the person must be able to subtract, multiply and divide, and perhaps most important, know which operation is appropriate.

This two-step process can be verified by recoding CRT responses into three categories (intuitive errors, non-intuitive errors, and non-intuitive correct responses) rather than the usual two categories of correct and incorrect. This additional information allows the separation of Cognitive Reflection (which distinguishes intuitive responses from non-intuitive ones) from numeric ability (which distinguishes non-intuitive correct responses from non-intuitive errors). Böckenholt (2012b) did this by treating cognitive reflection and numeric ability (labeled Inhibitory Control and Deliberate, respectively, in that paper) as separate latent variables in an item response theory model. This model fit better than a model with a single latent variable that was responsible for both checking the intuition and getting the correct answer (i.e., the simpler model effectively allowed only correct and incorrect responses). He also showed an hypothesized diurnal effect on cognitive reflection vs. numeric ability. In particular, morning people showed greater cognitive reflection in the morning than the evening, whereas evening people showed the opposite pattern (see also Bodenhausen, 1990). According to the author, no diurnal effect existed on the more trait-like (and presumably stable) numeric ability.

Campitelli and Gerrans (2014) produced a similar mathematical model and found that more cognitive reflection (labeled inhibition) was correlated with a greater likelihood to check intuitions in another cognitive bias: belief bias in syllogistic reasoning (Evans et al., 1983); their numeric ability construct (labeled mathematical computation) correlated with a three-item numeracy scale. However, they tested neither whether numeracy correlated with cognitive reflection nor whether belief bias correlated with numeric ability.

Although terminology and exact mathematical definitions of variables varied between the two studies, both studies conceptualized CRT responses as being comprised of cognitive reflection and numeric ability. In particular, Cognitive Reflection was the likelihood to give any non-intuitive answer and numeric ability was the conditional likelihood of giving the correct answer *given that* the answer was not intuitive.

## Do cognitive reflection and numeracy both predict good decision making?

Although studies have demonstrated correlations of the CRT with decision biases, it is unclear whether the effects are due to cognitive reflection (as usually posited) or numeric ability. Studies that separate cognitive reflection and numeric ability have not examined which is responsible for the CRT's relations with decision-making biases and outcomes. Two opposing hypotheses exist:

1. The Cognitive Reflection Hypothesis: Cognitive reflection will be responsible for the CRT's correlations with decision-making abilities. Numeric ability will not account for this relation.

However, cognitive reflection may only be predictive of decision making inasmuch as it correlates with numeric ability. In the present studies, we also examined performance of the Weller et al. (2013) numeracy scale. Because numeric ability may be a multi-faceted construct (Liberali et al., 2012; Weller et al., 2013) and the numeric skills required to solve CRT items are different from those tested on most numeracy scales, it is possible that the two numeric ability scales will account for different aspects of decision performance.

2. The Numeracy Hypothesis: Numeric ability measured on a numeracy scale and/or the CRT will account for the effects of cognitive reflection.

To test these hypotheses, we examined decision-making competence in two studies. To do so, we first used participants' CRT responses to identify separate constructs of Cognitive Reflection and numeric ability through cognitive modeling and/or the proportions of responses falling into the three categories described above (intuitive errors, non-intuitive errors, and non-intuitive correct responses). We then examined the relations of these constructs of Cognitive Reflection and numeric ability with good decision making. In Studies 1 and 2, we predicted consistency in risk perception from Bruine de Bruin et al.'s 2007 Adult Decision Making Competence (ADMC) scale. In Study 2, we also examined relations with under/overconfidence (Bruine de Bruin et al., 2007), performance on incentivized risky gambles and intertemporal preferences (Frederick, 2005), and self-reported financial outcomes. In both studies, we considered whether a standard numeracy scale could account for any findings and used large, diverse samples. We focused on testing whether the Cognitive Reflection Hypothesis or the Numeracy Hypothesis provided the best explanation of the data.

## Study 1

According to the Cognitive Reflection Hypothesis, greater cognitive reflection allows people to check faulty intuitions and, thus, reduce decision biases. Alternatively, the Numeracy Hypothesis posits that a lack of numeric ability produces these same biases. In the present study, we tested whether CRT performance was a significant predictor of decision biases due to Cognitive Reflection or numeric ability (called Calculation from here on when it is estimated from CRT responses). In Study 1, we attempted to find a bias that might be better predicted by Calculation rather than Cognitive Reflection. Consistent with the Numeracy Hypothesis, Del Missier et al. (2012) had found that consistency in risk perception (Bruine de Bruin et al., 2007) was predicted by numeracy, but not performance on inhibition tasks like the Stroop test. Although they did not test whether the CRT *per se* was predictive of consistency in risk perception, they did find that numeracy and inhibition independently predicted scores on the CRT (Del Missier et al., 2012). Consistency in risk perception was therefore a good candidate task.

### Methods

#### Participants and procedure

As part of the Understanding America Study, data were collected over the internet from a diverse sample (*N* = 1413) from 5/31/14 to 10/22/14. Data collection was approved by the Institutional Review board of the University of Southern California. An address-based sampling method was used to recruit participants. Participants completed financial literacy questions, personality questions, the risk consistency subscale of the ADMC, and, finally, numeracy. Financial literacy and personality will not be discussed in the present paper. Participants were paid $10 to complete the survey which took, on average, about half an hour.

#### Materials

##### Consistency in risk perception

In the consistency in risk perception subscale of the ADMC, participants were asked to estimate the likelihood of a number of events (e.g., getting in a car accident) happening to them in the next year on a scale of 0–100%. The events are set up in such a way that participants can commit framing inconsistencies as well as conjunction inconsistencies for subset/superset relations and time (see below). Note that in the present study, we separated the three types of risk consistency scores because they correlated only modestly and are predicted by different variables (especially the time conjunction score) as described below.

*Framing inconsistency*. Some of the events were complementary. The framing inconsistency score was the number of pairs of complementary items (out of four possible pairs) on which the sum of provided likelihoods was 10 or more points away from 100 (we introduced this threshold in order not to penalize participants who used more precise values; results were similar with other thresholds, including 5, 15, and 20; the 10 threshold worked best for the Cognitive Reflection Hypothesis and was retained). For example, if a participant indicated that his likelihood to drive accident free for the next 5 years was 80% and his likelihood to get into an accident in the next 5 years was 40%, then he would be scored as inconsistent for this pair of items.

*Subset/superset and time conjunction fallacies*. Some events were subsets of other events, for example, going to the dentist to fill a cavity was a subset of going to the dentist for any reason. The first conjunction fallacy (subset/superset) score was the number of times a subset event was judged as more likely than a superset event (out of four possible pairs). For example, if a participant indicated that her chance to go to a dentist in the next 5 years for any reason was 60%, and her chance to go to a dentist in the next 5 years to fill a cavity was 70%, then she would be scored as inconsistent for this pair of items.

The second conjunction fallacy (time) score was the number of times an event happening in the next year was judged as more likely than the same event happening in the next 5 years (out of 8 possible pairs). For example, if a participant indicated that is chance to go to the dentist in the next 5 years was 60% and his chance to go to the dentist in the next year was 70%, then he would be scored as inconsistent for this pair of items.

##### Numeracy and CRT

Participants completed the 8-item Rasch-based numeracy scale (Weller et al., 2013), which includes two CRT items. Participants also completed three additional CRT items (Toplak et al., 2014). Numeracy was scored as the proportion of non-CRT numeracy items answered correctly (out of a possible six items). Numeracy was mean-centered and standardized to match the scales of Cognitive Reflection and Calculation, which were estimated and scored as latent variables as described below, and as proportions. Cognitive Reflection was calculated as the proportion of CRT responses that were not the intuitive response (but they could be correct or incorrect; α = 0.48). Calculation was computed as the proportion of non-intuitive CRT responses that were correct (i.e., it is the conditional probability of answering correctly *given that* the participant answered non-intuitively).

#### Analyses

We estimated a model identical to Böckenholt's (2012b) Cognitive Miser model of the CRT. Their approach (unlike that of Campitelli and Gerrans, 2014) allows the estimation of individual differences and differences between items, accounts for measurement error, and allows the two abilities to be correlated. It is theoretically grounded in the Item Response Theory tradition. We used the nlme package Version 3.1 for linear and non-linear mixed-effects models (Pinheiro et al., 2014) to fit Böckenholt's model because it handles missing observations and allows for dichotomous response variables. De Boeck and Partchev (2012) described in detail how a package for generalized linear mixed-effects models can be used to fit an IRTree model, of which the Cognitive Miser model is one example (see also Böckenholt, 2012a). We describe this method briefly below.

Responses to the five CRT items were treated as up to 10 repeated measures because it is assumed that participants complete a two-step process when answering a CRT problem. To respond correctly, they must successfully complete both steps. In Step A, they attempt to avoid the intuitive response; if they fail to avoid it, processing is terminated and the incorrect intuitive response is given. If they avoid the intuitive response, then participants proceed to Step B and determine a non-intuitive response. If a participant reported the incorrect intuitive response, the process was assumed to have terminated in Step A. Thus, Step B was never performed, and the Step B response was treated as missing data. See Table 1 for a depiction of how data were coded. We used model comparisons to test an hypothesis concerning whether two separate abilities (vs. a single ability) were responsible for completing steps A and B.

**Table 1:** **Coding of possible responses**.

**Response**	**Step 1**	**Step 2**
Intuitive error	0	Missing
Non-intuitive correct	1	1
Non-intuitive error	1	0

##### Model 1

In the first model, we allowed only one factor to be responsible for individual differences in answering correctly on each step of each question, but estimated the population level difficulty for each step of each question. Based on this model's constraint of having only one factor responsible for individual differences, if subject 1 is twice as likely to be correct on step A for a problem as subject 2, she must be twice as likely as subject 2 to be correct on that same problem's step B, the next problem's step A, etc. However, although the model constrains individual differences, it does allow step A in one problem to be more or less difficult than step B for the same problem which can be more or less difficult than step A for the next problem, etc. Hence, this model has 10 fixed effects: five coefficients for the difficulty of step A (one for each of five problems) and five coefficients for the difficulty of step B. In addition, it has one source of variation between people (random effect).

##### Model 2

As in Model 1, population-level differences in difficulty still exist between all the repeated measures. However, in Model 2, we allowed two abilities to explain sources of individual differences - one for step A (Cognitive Reflection) and the other for step B (Calculation). In this model, if subject 1 is twice as likely to be correct on step A of the first problem as subject 2, he is not necessarily twice as likely to be correct as subject 2 on step B of the same problem, but is still twice as likely to be correct as subject 2 on step A of the next problem. The correlation between Cognitive Reflection and Calculation was estimated; hence, Step A performance may or may not influence performance on Step B. This model has the same 10 fixed effects as Model 1, but it has two individual difference parameters: (σ _Cognitive Reflection_, σ _Calculation_), and one parameter for the correlation between these abilities (γ). If this model fits better than the first model, we can conclude that two separate abilities influence CRT responses.

### Results

#### Identifying inattentive participants

Inattentive participants would be counted as high on Cognitive Reflection because their nonsensical CRT responses would count as non-intuitive. We found and excluded four participants whose numeracy responses displayed a non-sensical pattern (e.g., entering 10 or 100 for most questions). Removing these participants did not substantially alter the results (results including these participants are available from the first author).

These exclusion criteria could be considered conservative, meaning that some inattentive participants may have given responses that did not exhibit a clear pattern. To allow for this possibility, we conducted robust regressions (available in the Appendix). These results mirror the results reported in the main text, but account for the possibility that a relatively small portion of the sample may score high on Cognitive Reflection and have large decision biases, whereas the trend in the rest of the sample is the opposite. The similarity of these robust regressions to the results reported in the main text makes it unlikely that a relatively small group of inattentive participants influenced our results.

#### Descriptive statistics

The median participant earned between $50,000 and $60,000, was 49 years old, and had an associate degree; 52% of participants were female. See Table 2 for the proportion of participants giving each type of response on each item and Table 3 for means, standard deviations, correlations, and reliabilities of all scales.

**Table 2:** **Intuitive and correct responses for CRT items used in Study 1**.

**Problem**	**Responses**			**Proportion of responses that are:**		
	**Intuitive error**	**Correct**	**Common other error**	**Intuitive**	**Correct**	**Other error**
A bat and a ball cost $1.10 in total. The bat costs $1.00 more than the ball. How much does the ball cost? (in cents)	10	5	1, 105	78%	14%	8%
In a lake, there is a patch of lily pads. Every day, the patch doubles in size. If it takes 48 days for the patch to cover the entire lake, how long would it take for the patch to cover half of the lake?	24	47	12, 96	54%	29%	17%
Jerry received both the 15th highest and the 15th lowest mark in the class. How many students are in the class?	15, 30^*^	29	1, 35	20%, 47%^*^	18%	15%
A man buys a pig for $60, sells it for $70, buys it back for $80, and sells it finally for $90. How much has he made?	10	20	0, 30	43%	31%	26%
Simon decided to invest $8000 in the stock market 1 day early in 2008. Six months after he invested, on July 17, the stocks he had purchased were down 50%. Fortunately for Simon, from July 17 to October 17, the stocks he had purchased went up 75%. At this point, Simon has: (a) broken even in the stock market, (b) is ahead of where he began, (c) has lost money	B	C	A	43%	47%	10%
		Overall		57.0%	27.8%	15.3%

* *The class grades question has two possible intuitive errors (15 and 30), both of which are quite common. Results are similar if one or both errors are counted as the intuitive error; both errors were counted as intuitive errors for purposes of the present paper*.

**Table 3:** **Correlations of the measures in Study 1**.

	**Numeracy**	**Cognitive Reflection**	**Calculation**	**Frame Inconsistency**	**Conjunction (Sets)**	**Conjunction (Time)**
Cognitive Reflection	0.46					
Calculation	0.57	0.67				
Frame Inconsistency	−0.24	−0.20	−0.25			
Conjunction (Sets)	−0.23	−0.09	−0.15	0.23		
Conjunction (Time)	−0.05	−0.03	−0.08	0.20	0.17	
Mean	0	−0.01	0.04	1.27	0.40	1.83
*SD*	1	0.86	1.27	1.10	0.66	1.20
Reliability (alpha)	0.67	0.54	–	0.43	0.04	0.37

*All correlations were significant at the 0.05 level except Conjunction (Time) with numeracy (p = 0.07) and Cognitive Reflection (p = 0.37). The alpha for Cognitive Reflection represents the unstandardized Cronbach's alpha for the number of items that were answered with any non-intuitive response. Alpha for Calculation cannot be calculated because this variable is either a latent variable (Study 1) or a proportion with a variable denominator (Studies 1 and 2)*.

#### Modeling

Model 2 fit the data substantially better than Model 1 (change in BIC = 198; χ^2^_(2)_ = 217, *p* < 0.001), replicating previous results (Böckenholt, 2012b; Campitelli and Gerrans, 2014). Model 1 results consisted of the same fixed effects as in Model 2, but estimated less accurately. Therefore, we report only the results of Model 2.

Calculation varied more in the sample (σ _Calculation_ = 2.1) than did Cognitive Reflection (σ _Cognitive Reflection_ = 1.2), suggestive of the CRT measuring individual differences in Calculation to a larger degree than Cognitive Reflection. This is similar to Calculation having a higher reliability than Cognitive Reflection in a traditional analysis (Cronbach's alpha for Calculation cannot be calculated because this variable is either a latent variable or a proportion with a variable denominator). The two abilities correlated substantially (γ = 0.40) similar to γ = 0.31, calculated from the variances and covariances provided by Böckenholt (2012b); a correlation could not be computed for Campitelli and Gerrans' model as it does not estimate variances or covariances. We also found substantial differences in difficulty in both Cognitive Reflection and Calculation among the items (see Table 4). Coefficients in the table indicate log odds, so a coefficient of 0 indicates that participants were, on average, as likely to do the task correctly as they were to fail; higher coefficients indicate greater chances of doing the task correctly (e.g., 0.3 indicates the odds of answering correctly vs. incorrectly are *e*^0.3^ = 1.35, and the probability of answering correctly is 0.57). Consistent with Frederick (2005), Calculation was easier than Cognitive Reflection; however, Calculation was far from trivial. For example, in the Bat and Ball problem, Calculation (β = 0.32) was substantially easier than Cognitive Reflection (β = −1.64); however, people still failed to calculate correctly almost half the time. Calculation in the investment problem, on the other hand, was quite easy (β = 2.10). This is sensible because the investment problem is multiple choice so that simply eliminating the intuitive option narrows the set of choices to only two possibilities.

**Table 4:** **Model difficulty parameters (standard errors) for each CRT item**.

**Item**	**Cognitive Reflection**	**Calculation**
Bat and ball	−1.65 (0.08)	0.32 (0.18)
Lily pad	−0.23 (0.07)	0.23 (0.12)
Class size	−0.89 (0.07)	−0.28 (0.14)
Pig sale	0.38 (0.07)	0.03 (0.11)
Investment	0.37 (0.07)	2.10 (0.14)

To determine how Cognitive Reflection and Calculation related to numeracy and decision performance, we estimated the random effects of these variables by participant. These random effects are the modes of the distributions of Cognitive Reflection and Calculation conditional on the model for each participant (i.e., the most likely Cognitive Reflection and Calculation scores given that Model 2 is correct). In other words, Cognitive Reflection and Calculation, as discussed below, are scores for these constructs for each participant derived from the model. As expected, we found that greater numeracy was correlated with greater Calculation (*r* = 0.57, *p* < 0.001), replicating Campitelli and Gerrans (2014) finding. However, we also found that Cognitive Reflection had roughly the same correlation with numeracy (*r* = 0.46, *p* < 0.001). Greater Calculation was also correlated with greater Cognitive Reflection (*r* = 0.67, *p* < 0.001); the correlation explicitly estimated in the model, γ = 0.40, is likely more reliable. This correlation may reflect a general ability like intelligence, or something more specific to performance on CRT problems.

Since Cognitive Reflection and Calculation were correlated, and each was substantially correlated with numeracy, we conducted multiple regressions for each decision bias to partial out shared variance and, hence, to test which part of the CRT independently predicted biases (see Table 5). We examined the participants who completed all the tasks: consistency in risk perception subscale, numeracy, and CRT (final *N* = 1225). Analyses conducted on all participants who completed each subscale produced similar results as did robust regression analyses (see Appendix) and multiple regressions using Cognitive Reflection and Calculation scores computed as proportions^1^.

**Table 5:** **Regression analyses in Study 1—Consistency in risk perception and CRT**.

	**Frame Inconsistency**		**Conjunction (subset vs. superset)**		**Conjunction (time)**	
	**Without numeracy**	**With numeracy**	**Without numeracy**	**With numeracy**	**Without numeracy**	**With numeracy**
Intercept	**2.14 (0.20)**	**2.04 (0.20)**	**0.98 (0.12)**	**0.87 (0.12)**	**2.11 (0.23)**	**2.11 (0.23)**
Cognitive Reflection	−0.07 (0.05)	−0.06 (0.05)	0.04 (0.03)	0.05 (0.03)	0.08 (0.06)	0.08 (0.06)
Calculation	**−0.12 (0.03)**	**−0.09 (0.03)**	**−0.05 (0.02)**	−0.02 (0.02)	**−0.10 (0.04)**	**−0.10 (0.04)**
Numeracy	–	**−0.13 (0.04)**	–	**−0.11 (0.02)**	–	0.00 (0.05)
F	**20.4**	**19.1**	**12.7**	**14.0**	**2.97**	**2.54**
df	6, 1218	7, 1217	6, 1218	7, 1217	6, 1218	7, 1217
R^2^	0.09	0.09	0.05	0.07	0.01	0.01

*Each dependent variable was regressed onto Cognitive Reflection and Calculation and all demographic variables (age, income, education, and gender), though their coefficients were not reported for simplicity. The results were reported in the columns titled “Without Numeracy.” Numeracy was then added and the results were reported in the columns titled “With Numeracy.” Values are unstandardized beta coefficients with standard errors in parentheses. Bold font denotes statistical significance at p < 0.05*.

#### Frame inconsistency

In multiple regression with frame inconsistency as the dependent variable, we found that higher Calculation (*p* < 0.001) but not Cognitive Reflection (*p* = 0.15) independently predicted less frame inconsistency (e.g., more consistency between estimated likelihoods to drive accident free vs. get into an accident) after accounting for demographic variables. When numeracy was added to the model (*p* = 0.001), it added significant independent predictive power and did not completely account for the variance explained by Calculation (*p* = 0.01).

#### Conjunction fallacies (subset vs. superset; time)

Greater Calculation (*p* = 0.01), but not Cognitive Reflection (*p* = 0.13), predicted fewer conjunction fallacies between subset and superset events (e.g., participants estimated more consistent likelihoods between going to the dentist for any reason and going to the dentist to fill a cavity). When numeracy was added to the model (*p* < 0.001), it accounted for the variance explained by Calculation (*p* = 0.33). Calculation (*p* = 0.01) also predicted conjunction fallacies between points in time; Cognitive Reflection did not (*p* = 0.16). When numeracy was added to the model, it did not explain additional significant variance (*p* = 0.93), and it did not account for the effects of Calculation (*p* = 0.01). We examine time conjunction fallacies again in Study 2.

### Discussion

The present study replicated and extended earlier results from Campitelli and Gerrans (2014). In particular, Cognitive Reflection and Calculation behaved like distinct abilities, and Calculation was positively correlated with numeracy. However, Cognitive Reflection was positively correlated with numeracy as well; this correlation had not been tested in earlier studies. This finding, however, may not be surprising given that a numeric formula is needed to check the intuition in CRT problems (e.g., in the bat and ball problem, $1.00 + $0.10 + $0.10 ≠ $1.10). Thus, numeracy may be important to both steps in solving CRT problems; setting up a numeric formula is necessary to check intuitions and adequate numeric ability is necessary to solve the formula.

Contrary to the Cognitive Reflection Hypothesis, Cognitive Reflection did not provide any unique explanatory power in Study 1's decision tasks, whereas Calculation and numeracy did in both tasks. It was not entirely clear whether the non-significance of Cognitive Reflection in predictions of conjunction fallacies may have been due to numeric ability accounting for its effects or because it was not a potent predictor in the first place. Our model also showed that the CRT measures Calculation to a greater degree than it measures Cognitive Reflection. Therefore, our results could be explained in part by Calculation's relatively low reliability (though its reliability was not much lower than that of numeracy). This is important because it suggests that previous results that attribute the predictiveness of the CRT to a cognitive reflection construct may be in error, given that the scale measures Calculation to a greater degree. Our results were most consistent with the Numeracy Hypothesis although we had not expected Calculation to be predictive beyond numeracy. We offer a possible explanation in the general discussion. The dependent measures in this study were derived from a subscale of decision making competence, consistency in risk perception, which we expected to correlate with Cognitive Reflection but be explained by numeric ability (Bruine de Bruin et al., 2007; Del Missier et al., 2012).

Note that conjunction fallacies regarding time had unacceptable reliability, as measured by Cronbach's alpha, even compared to the relatively low reliability of the other decision biases. However, conjunction errors about time correlated substantially with our predictors and the framing bias. The fact that the reliability was lower than the variance explained in our models suggests that either our results were due to chance or that Cronbach's alpha measure of reliability may not be appropriate, perhaps because it is an estimate of the lower bound of reliability (Cronbach and Shavelson, 2004). These low reliabilities point to the need to replicate the present results. In Study 2, we attempted to replicate our results, but also turned to tasks that have been related to CRT performance more traditionally in past research. We also examined directly incentivized tasks and more real-world decision outcomes.

## Study 2

In Study 2, we again examined the ADMC's consistency in risk perception, but we also focused on decision tasks more traditionally associated with cognitive reflection. In particular, we examined under/overconfidence (another subscale of the ADMC). Hoppe and Kusterer (2011) demonstrated that correct levels of confidence were correlated with higher CRT scores (see Del Missier et al., 2012 for similar results with a presumably related inhibition measure). However, other research suggests that numeracy may be independently predictive as well (Winman et al., 2014). We also examined intertemporal and risky choices similar to those originally studied by Frederick (2005). The Cognitive Reflection Hypothesis suggests that the CRT's predictive ability in these tasks is due to cognitive reflection, not numeric ability. Consistent with the Numeracy Hypothesis, however, research has demonstrated that greater numeracy is related both to more patience in intertemporal choice and more expected-value-consistent risky choices (Benjamin et al., 2013). In addition, we examined whether CRT and/or numeracy would be associated with inconsistent responses in risky choices. In particular, we expected that lower numeracy or worse CRT performance would be associated with risky choices that were logically inconsistent with previously expressed preferences. No previous studies have considered CRT or numeracy relations with this inconsistency.

Finally, we examined self-reported financial outcomes and predicted that both cognitive reflection and numeric ability would independently predict having retirement savings, paying bills on time, and not taking predatory loans. Avoiding undesirable financial outcomes likely requires understanding how costly bad financial moves can be; less numerate individuals do not fare well in this regard (Soll et al., 2013). It also may require self-regulation (related to cognitive reflection by Böckenholt, 2012b) to control impulsive spending (Vohs and Faber, 2003). Thus, we expected that both numeric ability and Cognitive Reflection would independently predict positive financial outcomes.

### Methods

#### Procedure

Participants in RAND's American Life Panel (ALP: http://www.rand.org/labor/alp.html) were paid $20 to complete each half hour Internet survey. Data collection was conducted and approved by RAND Corporation. The various questionnaires described below were administered at different points in time. A total of 1478 participants provided demographic information and responses to CRT and other numeracy items. Stepwise regressions to predict the decision bias composite were conducted on the 939 participants who completed those items and at least one each of the intelligence measures and decision-bias tasks. Stepwise regressions to predict the financial outcome composite were conducted on the 1131 participants who completed demographics, numeracy, CRT, and at least one each of the intelligence and financial-outcome tasks.

#### Measures

We examined the same ADMC subscales as in Study 1 and several additional decision making tasks. Participants also completed the Weller et al. (2013) numeracy scale and an additional CRT item.

##### Consistency in risk perception

A complete version of the consistency in risk perception scale was administered and scored in the same way as in Study 1. The scale included four pairs of framing inconsistency pairs, six subset/superset conjunction pairs, and 10 time conjunction pairs.

##### Under/over/accurate confidence

Participants were asked if they thought fourteen general knowledge statements (e.g., “Amman is the capital of Jordan”) were true or false, and they indicated their confidence that they answered each item correctly from 50% (just guessing) to 100% (absolutely sure). We used the absolute difference between the percentage of items answered correctly and the average confidence across items to assess confidence accuracy.

##### Incentivized intertemporal choice

Participants were asked if they wanted their payment for the survey to be mailed immediately, or 110% of those payments to be mailed 10 days later. Participants were shown the amounts they would be mailed in each case and were rewarded according to the plans they chose. This variable was coded 0 (indicating a preference for more money later) or 1 (indicating a preference for less money now).

##### Incentivized risky choice

The Holt-Laury Procedure was employed (Holt and Laury, 2002). Specifically, participants were asked their preferences between ten pairs of gambles, all in the domain of gains. Each pair of gambles included one safe gamble, in which the participant could win either $2.00 with some probability, otherwise get $1.60, and one risky gamble, in which the participant could win $3.85 with the same probability, otherwise get $0.10. The response consistent with an expected-value calculation was the safe gamble when the probability was low (e.g., the gamble “10% chance to win $2.00, otherwise $1.60” has a higher expected value than the gamble “10% chance to win $3.85, otherwise $0.10”) and the risky gamble when the probability was high (e.g., “90% chance to win $2.00, otherwise $1.60” has a lower expected value than “90% chance to win $3.85, otherwise $0.10”). In the case of 100%, the “risky gamble” amounted to receiving $3.85 and dominated the “safe gamble,” which amounted to receiving $2.00. Each participant was given a risk aversion score, which was the number of times the safe gamble was chosen. One of the choices was played for real, and any payoff was added to the participant's survey payment.

Participants chose between the two gambles at 10 probabilities in the same fixed order (10, 20, 30,… 100%). Due to the fixed order, once the risky gamble was chosen, it should be preferred in all subsequent choices regardless of risk preferences because increasing the probability simply makes it better compared to the safe gamble. Hence, each participant was also given a consistency score, which was the minimum number of choices that had to be changed so that the participant would have consistent preferences.

##### Numeracy and CRT

Three items are too few for the latent variable modeling approach of Study 1, and the model Campitelli and Gerrans (2014) used was inappropriate because it is unable to produce individual scores for participants. Instead, Cognitive Reflection and Calculation were estimated using proportions (see Study 1). We chose this approach because it is conceptually similar to and more transparent than earlier modeling approaches (Böckenholt, 2012b; Campitelli and Gerrans, 2014). About 24% of participants answered all CRT problems intuitively, making their Calculation scores non-sensical^2^. We gave these participants Calculation scores of 0 although results were essentially identical if scores were instead imputed using linear fits from variables correlated with Calculation including numeracy, education, income and gender (Enders, 2010)^3^. The same 8-item numeracy scale as in Study 1 was administered, and six of its non-CRT items were used as a measure of numeracy (α = 0.58).

##### Financial outcomes

Participants reported five financial decision-making outcomes (see Table 6). Each was coded 0 if the outcome was unfavorable and 1 if favorable.

**Table 6:** **Financial outcomes in Study 2**.

**Name**	**Question**	**Answers counted as “success”**
Avoided predatory loans	Within the last year, have you obtained credit from a rent-to-own store, pawn shop, payday lender, cash advance lender, auto title lender, or tax return preparer?	No
Avoided being denied credit	Have you been denied credit for any type of loan within the last year?	No
Saved money for retirement	What is the total amount of wealth you have accumulated so far for the purpose of retirement preparation, including both accounts like 401 k or IRA and also any other types of accounts or forms of retirement saving?	Not 0
Loans on time	Have you made a late payment on any loan in the last year?	No
Paid credit cards in full^*^	Over the past 12 months, I always paid my credit cards in full	Yes

* *Only participants who said they had a credit card in the past 12 months (N = 1207) were asked about whether they paid it in full*.

##### Intelligence measures

Participants completed four non-numeric intelligence measures (Raven's Matrices, antonyms, a vocabulary measure that required identification of words from pictures, and verbal analogies). Scores indicated the number of questions answered correctly^4^. Scores on each test were standardized (i.e., divided by its standard deviation and mean centered) and averaged to derive a composite intelligence measure (standardized α = 0.48). If a score on a particular test was missing for a participant, only scores on the remaining tests were used to calculate that participant's composite score^5^.

##### Composites

To avoid testing our seven decision biases and five financial outcomes one at a time, we created two composites^6^ (see Toplak et al., 2011 for the use of composites in a similar context). A decision-bias composite was computed as the average of the standardized decision-bias variables (Framing Inconsistency, Conjunction (subset vs. superset), and Conjunction (time), Under/Overconfidence, Impatient Intertemporal Choice, Risk Aversion and Risk Inconsistency). Standardized alpha was low (0.37), but comparable to previous research (e.g., Toplak et al., 2011). Scores were standardized and averaged in a manner similar to the intelligence measures above. Thus, if a participant only completed the under/overconfidence measure, and scored 1 standard deviation (SD) higher than the mean, 1 would serve as his/her decision-bias score. But if that participant also completed the risk-preference choices and scored 1 SD lower than the mean on both risk aversion and choice inconsistencies, he would receive a score of (1-1-1)/3 = −1/3. A financial-outcome composite was computed as the number of positive financial outcomes divided by the number of financial-outcome questions answered.

### Results

#### Identifying inattentive participants

Eighteen (out of 1478) participants were deleted due to numeracy responses that followed a pattern indicating inattention (e.g., entering 10 or 100 for most questions). Their deletion did not significantly alter results.

#### Replications

As in Study 1, greater Cognitive Reflection was correlated with greater Calculation and both were correlated with higher numeracy (Table 7). We replicated Study 1's framing inconsistency results: Greater Cognitive Reflection was correlated with less bias (*r* = −0.12, *p* < 0.001). In multiple regression and after controlling for demographic variables, however, Calculation and numeracy accounted for the effects of Cognitive Reflection [regression coefficients are Calculation: *b* = −0.05, *p* = 0.07, numeracy: *b* = −0.16, *p* = 0.002, and Cognitive Reflection: *b* = −0.01; *p* = 0.81, final model *F*_(7, 918)_ = 6.07; *p* < 0.001; *R*^2^ = 0.04]. We also found that greater Cognitive Reflection was correlated with showing fewer conjunction fallacies between subset and superset events (*r* = −0.10, *p* = 0.002). Again, when Calculation and numeracy were added in multiple regression, they accounted for the effects of Cognitive reflection [regression coefficients are Calculation: *b* = −0.06, *p* = 0.28, numeracy: *b* = −0.35, *p* = 0.004, and Cognitive Reflection: *b* = −0.004; *p* = 0.96, final model *F*_(7, 880)_ = 4.3; *p* < 0.001; *R*^2^ = 0.03]. Only age and gender (and none of our cognitive predictors) were related to time conjunction fallacies.

**Table 7:**
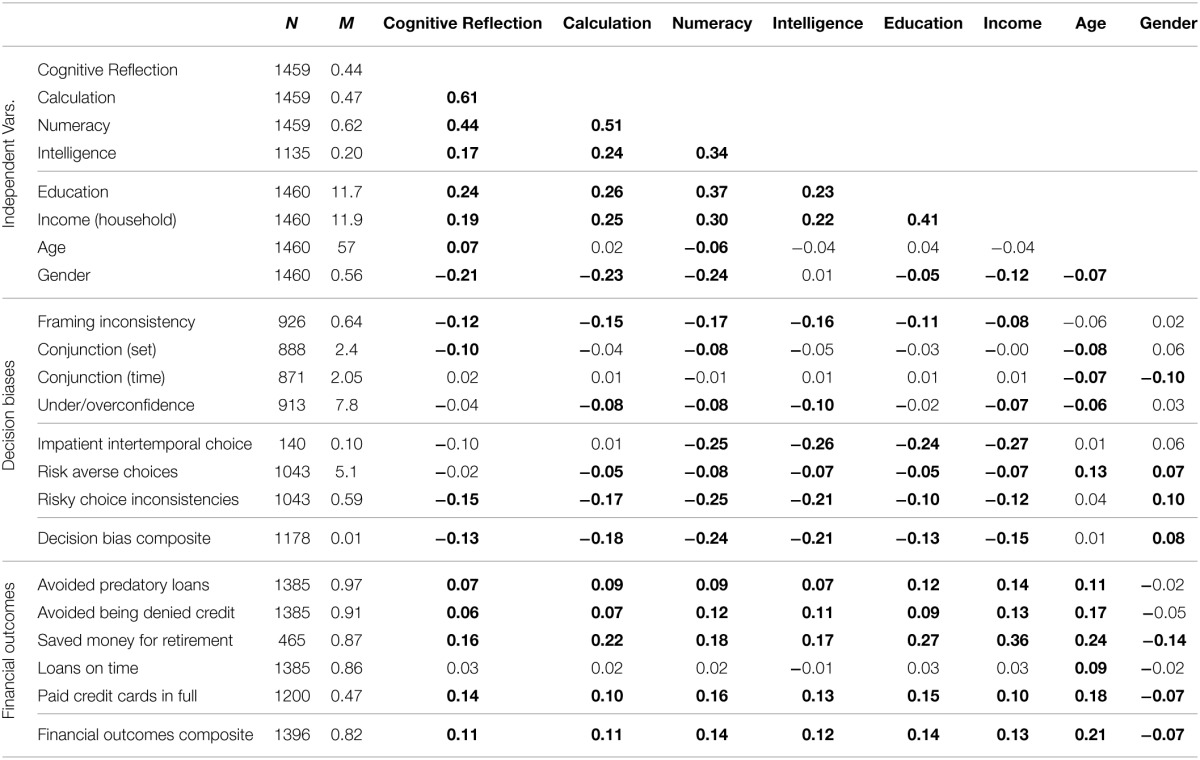
**Correlations of independent measures among themselves and with dependent measures in Study 2**.

*M, Mean; Bold indicates significance at p < 0.05. Gender was coded 0 (male) and 1 (female)*.

#### New decision biases and financial outcomes

Greater numeracy was related to showing less of each of the other decision biases, whereas greater Cognitive Reflection was only significantly related with fewer risky-choice inconsistencies (see Table 7). In multiple regression of risky choice inconsistencies, however, numeracy accounted for the effects of Cognitive Reflection [coefficients for numeracy, Calculation, and Cognitive Reflection were *b* = −0.77, *p* < 0.001, *b* = −0.09, *p* = 0.20 and *b* = −0.05, *p* = 0.57, respectively; final model *F*_(3, 1039)_ = 23.6; *p* < 0.001; *R*^2^ = 0.06]. Cognitive Reflection did not correlate with less risk aversion or more patient intertemporal choice (see Oechssler et al., 2009; Campitelli and Labollita, 2010 for similar results). Campitelli and Labollita (2010) also found that the CRT was related to more choices consistent with expected value. Similarly, we found that greater Cognitive Reflection correlated with more choices consistent with expected value (*r* = 0.15, *p* < 0.001). However, when numeracy and Calculation were added as predictors, Cognitive Reflection (*b* = −0.02, *p* = 0.91) and Calculation (*b* = 0.17, *p* = 0.27) became non-significant, whereas numeracy remained significant [*b* = 2.36, *p* < 0.001, *F*_(3, 1039)_ = 34.4, *p* < 0.001, *R*^2^ = 0.09]. Both Cognitive Reflection and numeracy correlated with each of the financial outcomes except making late loan payments.

As expected, we also found that greater intelligence, more education, greater income, younger age, and being male were correlated with greater Cognitive Reflection, Calculation, and numeracy. These potentially confounding variables were also correlated with decision biases and financial outcomes, possibly explaining the effects reported above. Thus, we conducted stepwise regressions to determine whether Cognitive Reflection and/or numeric ability retained independent predictive power above and beyond these variables.

#### Stepwise regressions

For both composites, we conducted a stepwise regression, adding variables in the following order: (1) gender, age, education, income, (2) intelligence composite, (3) Cognitive Reflection, (4) Calculation, and (5) numeracy. Full regression results are available in Table 8. In predictions of the decision-bias composite, demographic variables made little difference with the exception of greater income predicting fewer decision biases [model *F*_(4, 929)_ = 4.9, *p* < 0.001, *R*^2^ = 0.02]. Greater intelligence was related to less bias as expected (Stanovich and West, 1998; *b* = −0.21, *p* < 0.001; change in *R*^2^ = 0.02) and accounted for the effects of the demographic variables. Cognitive Reflection was a borderline significant predictor of decision biases beyond intelligence^7^ (*b* = −0.11, *p* = 0.052, change in *R*^2^ = 0.004). Greater Calculation was associated with fewer biases (*b* = −0.17, *p* < 0.001, change in *R*^2^ = 0.01) above and beyond IQ and Cognitive Reflection despite its high correlation with the latter; Calculation completely accounted for the effects of Cognitive Reflection. Numeracy was also a significant predictor of fewer biases (*b* = −0.42, *p* < 0.001, change in *R*^2^ = 0.02); it did not fully account for the effects of Calculation, which remained significant after numeracy was added [*b* = −0.12, *p* = 0.02; full model *F*_(8, 925)_ = 10.5, *p* < 0.001, *R*^2^ = 0.07].

**Table 8:**
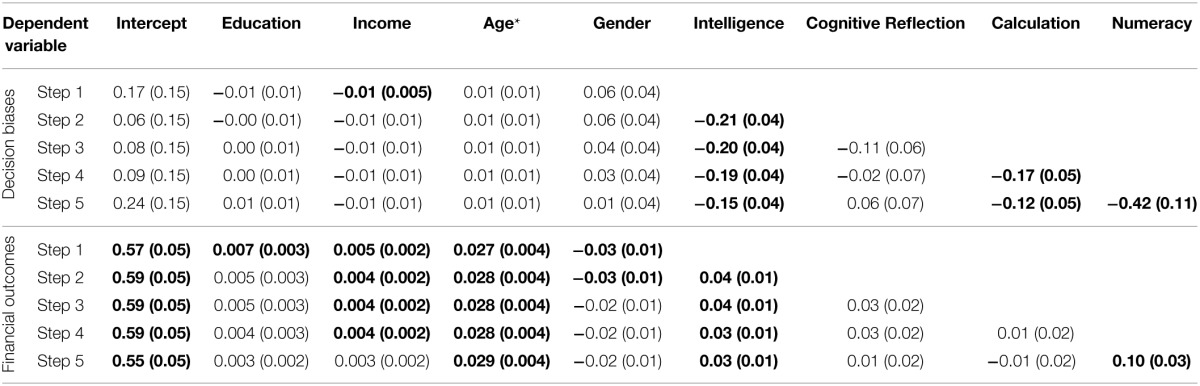
**Stepwise regression results predicting decision-bias and financial-outcome composites in Study 2**.

Unstandardized beta coefficients with standard errors in parentheses. Numeracy is the six-item numeracy scale used in both Studies 1 and 2. Bold font denotes statistical significance. ^*^Age was divided by 10 in this regression to make the coefficients more interpretable (effects of age are effects of being a decade older). Gender was coded 0 when male and 1 when female.

We predicted the financial-outcomes composite using a similar approach. In Step 1, demographic variables were predictive [model *F*_(4, 1118)_ = 18.6, *R*^2^ = 0.06], with more positive financial outcomes among those with greater education, income, and age. Higher intelligence was also predictive of better outcomes (*b* = 0.04, *p* = 0.002, change in *R*^2^ = 0.01). Cognitive Reflection was not a significant predictor of better financial outcomes (*b* = 0.03, *p* = 0.10, change in *R*^2^ = 0.002) above and beyond demographics and intelligence, even before accounting for Calculation and numeracy^8^. Calculation was also not significant (*b* = 0.01, *p* = 0.74, change in *R*^2^ < 0.001). In the final model, greater numeracy did predict better financial outcomes (*b* = 0.10, *p* = 0.004, change in *R*^2^ = 0.01) as did higher income and intelligence [full model *F*_(8, 1114)_ = 12.1, *p* < 0.001, *R*^2^ = 0.08].

### Discussion

As in Study 1, we found that numeric ability, not Cognitive Reflection, predicted framing inconsistencies and conjunction fallacies between subsets and supersets. We did not find any decision biases that Cognitive Reflection predicted independently. We also found that Calculation and numeracy, but not Cognitive Reflection, predicted a decision-bias composite that included subscales of the ADMC and the original two biases tested by Frederick (2005). In addition, only numeracy predicted financial outcomes independently. These results were inconsistent with the Cognitive Reflection Hypothesis and supported the Numeracy Hypothesis. Our findings cannot be explained by the high correlation between Cognitive Reflection and Calculation, since Cognitive Reflection was not predictive of either composite before Calculation was included in the model.

Numeracy was also related to less under/overconfidence (see also Winman et al., 2014). This finding is reasonable given that under/overconfidence is a task in which participants are asked to produce probabilities. However, contrary to expectations, Cognitive Reflection was not associated with more accuracy in this task. It may be that Hoppe and Kusterer's, (2011) finding that greater CRT scores were related to more confidence accuracy was due to them not separating Cognitive Reflection from Calculation.

Cognitive Reflection was also essentially uncorrelated with risk aversion and intertemporal choice in our experiment. This discrepancy from Frederick, 2005; findings may be because choices in our experiment were incentivized, but CRT responses were not. One study has shown that incentivized predictors are more strongly related to incentivized outcomes, at least in the case of beliefs predicting behavior (Gächter and Renner, 2010). Without incentives, Cognitive Reflection may be a skill that helps avoid biases and errors in low stakes situations but may be less relevant in predicting incentivized choices because everybody reflects sufficiently. Numeracy is a requirement to resolve a mathematical problem in any situation, possibly explaining why it is a better predictor of these outcomes across levels of incentives.

## General discussion

Results of the present studies were consistent with the CRT's role in decision-making biases and financial outcomes being due to numeric ability and not cognitive reflection. In addition, Study 1's model of CRT responses indicated that Calculation accounted for much more of the variance in responses than Cognitive Reflection did. These results are at odds with previous explanations invoking the importance of intuitions and labeled the Cognitive Reflection Hypothesis in the present paper (Kahneman, 2003, 2011; Frederick, 2005; Toplak et al., 2011). The CRT either is not an effective measure of the hypothesized ability to check and correct intuitions or this ability does not play a role independent of numeric ability in the decision biases we examined. It is possible that Cognitive Reflection does play a role in other biases, such as probability matching, shown to be related to the CRT in previous literature. The present results, however, support the Numeracy Hypothesis, which posits that individuals with greater numeric ability will demonstrate fewer decision biases and achieve better financial outcomes, and it will account for the predictive power of Cognitive Reflection.

The three-item CRT scale remains a quick-to-administer predictor of a number of decision-making biases. It is also interesting psychologically. Analyzing the cognitive reflection aspect of this scale continues to lead to insights about human reasoning almost 10 years after publication of the initial paper (e.g., De Neys et al., 2013; Mastrogiorgio and Petracca, 2014). In addition, the fact that there are detectable individual differences in Cognitive Reflection, that are somewhat stable across problems, may support the idea that individual differences in System 1 inhibition exist (Kahneman and Frederick, 2002; Frederick, 2005). These individual differences also may be related to executive inhibition, which itself relates to decision making in the lab (e.g., Del Missier et al., 2012) and in real life (e.g., Nigg et al., 2006; Roca et al., 2008). Theoretically, however, executive inhibition is distinct from Cognitive Reflection. The former measures the ability to inhibit a response, once it is clear that a response must be inhibited. The latter measures the ability to realize that a response should be inhibited in the first place (Toplak et al., 2011). Imagine two people choosing between a risky and an uncertain prospect of a higher expected value. The first chooses the risky option simply because he doesn't like the feeling of uncertainty. The second reflects that the uncertain prospect is objectively a better choice, but chooses the risky option nonetheless. The first person is likely low on Cognitive Reflection, whereas the second is likely low on inhibition, but not on Cognitive Reflection. They are also distinct empirically; in particular, Toplak et al. (2011) found that CRT scores explain more of the variance in heuristics and biases than does inhibition (they did not control for numeric ability).

One possible reason for Cognitive Reflection not being a potent predictor is its lower reliability compared to Calculation. The latent variable associated with Cognitive Reflection accounted for just over half the variance as did the latent variable associated with Calculation, indicating that inhibition of a default response on one item was not strongly related to inhibition of a default response on another item (i.e., not very reliable). This is problematic for the CRT, because sum scores, which are often used as the outcome measure for the CRT, likely measure the more reliable Calculation construct to a larger degree than Cognitive Reflection. It also indicates that scoring schemes that differentiate only between intuitive and non-intuitive responses may ignore much of the useful variance (see also Pennycook et al., 2015). However, this fact does not completely account for our results, since Cognitive Reflection has simple correlations with many of our dependent measures that then are accounted for by other factors (especially numeric ability) and its reliability was not much lower than that of numeracy (their respective Cronbach's alphas were 0.54 and 0.65 in Study 1).

Based on the present results, correlations with CRT scores appear insufficient for establishing a prominent role for checking intuitions, at least in the decision tasks we examined and contrary to prominent citations of such correlations in support of this role (Thaler and Sunstein, 2008; Kahneman, 2011). Instead, the CRT scale appears to measure multiple constructs. At least two approaches exist to resolving the issue of multiple constructs: (1) Separate the hypothesized components of the scale mathematically or (2) use scales that measure only one construct at a time. The first approach was taken in the present two studies; it requires careful analysis of inattentive participants because component scores can be muddied by inattentive participants. In each of our studies, removal of these few participants (about 1%) did not significantly influence our results. Lower quality convenience samples often suffer from large proportions of inattentive participants, however (e.g., Oppenheimer et al., 2009; Maniaci and Rogge, 2014), and such participants would be especially harmful to the Cognitive Reflection subscale. Techniques like robust regression can be used to automatically deal with such participants.

However, to attain a more pure measure of Cognitive Reflection, the second approach may be best: Scales that do not require the use of numeric skills should be used. Such scales would likely be less correlated with numeracy, and it would be interesting to see if they were uncorrelated when accounting for general intelligence. These scales could use problems that elicit an initial incorrect intuition but are not mathematical. Baron et al. (2014), for example, used syllogisms for this purpose.

The problem of multiple constructs may also apply to numeracy scales that include the original three CRT items (e.g., Weller et al., 2013). Performance on decision-making tasks may correlate with this numeracy scale due to Cognitive Reflection. However, the present results do not support this idea. In addition, four of five published studies of numeracy and CRT items supported them being part of a single numeric ability using factor analytic techniques (Liberali et al., 2012, Study 1; Weller et al., 2013; Baron et al., 2014; Låg et al., 2014).

Another issue exists, however, both for the CRT and for numeracy scales that include CRT items. The original three CRT items have been well publicized; they are commonly administered in internet surveys, have shown up in newspaper articles and radio shows, and are shown to undergraduates in courses. Problematically, experience with these problems is known to increase later performance (Chandler et al., 2014), and it may test memory rather than performance. Indeed, studies recently conducted online show unusually high performance on the CRT (e.g., Mastrogiorgio and Petracca, 2014). In the present samples, it was unlikely that participants had prior exposure to the CRT items because Study 1 used mostly new CRT items (Toplak et al., 2014) and Study 2 took place in 2006, not long after the CRT was first published. In future studies, new CRT problems can be used, but it is currently unknown whether similar practice effects may exist with these new problems. In addition, because non-CRT numeracy problems were more potent predictors of decision biases and financial outcomes anyway, the best approach for future research may be to use these non-CRT numeracy items and systematically vary them while retaining similar difficulty levels.

One surprising finding was that, in both studies, numeracy did not fully account for the effects of Calculation in predicting decision-making biases. It may be that CRT Calculation indexes an aspect of numeracy, like algebraic ability, that was not otherwise measured in the Weller et al. (2013) numeracy scale. Alternatively, it may simply be, as Weller et al. found, that the remaining numeracy items were easier than CRT Calculation and that the added difficulty teases apart additional variance in decision biases among the most numerically able. Numeracy scales that separate various aspects of numeric ability may be useful (Ghazal et al., 2014; see Weller et al., 2013 for discussion).

The proportions of variance explained in our studies were low to moderate (e.g., *R*^2^= 0.07 for the final model predicting Study 2's financial outcomes). These results may be due, at least in part, to the low reliabilities of the composite measures we used as dependent variables (though our reliabilities were not much lower than those of studies with similarly broad decision-making bias composites, e.g., Toplak et al., 2011). In addition, however, our composite measures represented multiple constructs. Individual items varied in how well they were predicted (see the simple correlations of Table 7). Although explaining more variance and having more reliable scales would indeed be desirable, numeracy nonetheless was an important predictor of decision biases and financial outcomes even after controlling for other variables (e.g., cognitive reflection, income). For example, although most people experienced primarily good financial outcomes (Mean = 82% of good financial outcomes, Median = 80%), a person who correctly answered one out of the 6 possible numeracy items was predicted to have 78.5% positive outcomes, whereas a person who scored 5 out of 6 correct was predicted to have 85.0% positive outcomes; this difference was enough to move a person from the 25th to the 65th percentile of financial outcomes in our sample. Given the crucial role these outcomes can play in life, the difference may be important. More important to the focus of the present paper, the data allow us to examine composites and biases for which Cognitive Reflection is a statistically robust predictor in simple correlations, but lacks any significant predictive power in the presence of numeracy.

Substantial research exists indicating that the CRT correlates with decision-making biases; various authors claim either cognitive reflection or general intelligence as explanations. The present results point instead to numeracy as a more important explanatory construct. Future research should examine what specific aspects of numeracy matter to what kinds of decision making. For example, is knowing simple arithmetic sufficient? Peters et al. (2010), for example, used simple arithmetic problems to assess numeracy among Ghanaian villagers who did not know what abstract probabilities were; greater numeracy was associated with better decision-task performance and taking more protective health behaviors against HIV. Perhaps, one must also know certain mathematical strategies and definitions for better decision making competence in some domains.

Peters and Bjalkebring (2014) suggested instead there are fundamentally different ways of knowing numbers. In particular, judgments and decisions are multiply determined by objective numeracy (associated with explicit number operations such as number comparisons and calculation), subjective numeracy (linked with motivation and confidence with the use of numbers), and number intuitions (the mapping of symbolic numbers onto magnitude representations was associated with numeric memory and valuation processes). Subjective numeracy (Fagerlin et al., 2007), beliefs in one's own mathematical competence, may be a particularly overlooked measure. Being willing to work with numbers in decisions may distinguish between competent and incompetent decision makers more than being able to do the math (Peters and Bjalkebring, 2014).

CRT research has been conducted primarily in the lab and is subject, therefore, to concerns about external validity. The same is true (although less so) for numeracy. The present research demonstrates, however, that CRT Calculation, as well as numeracy, predict decision-making competence in the lab and in real world outcomes for diverse samples of people.

### Conflict of interest statement

The authors declare that the research was conducted in the absence of any commercial or financial relationships that could be construed as a potential conflict of interest.

## Figures and Tables

**Table A2:**
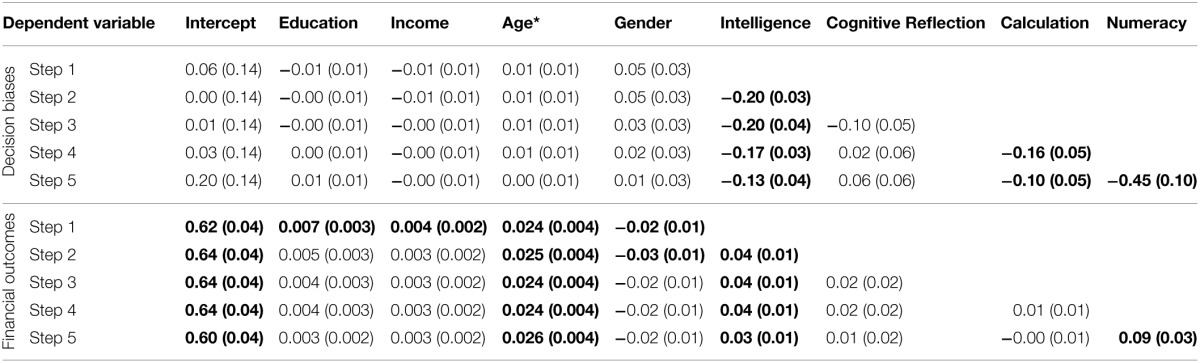
**Robust regression results from Study 2 (see Table 8 for linear regression results)**.

## Footnotes

^1^These estimates are subject to measurement error and, in the case of Calculation, are heteroskedastic. Nonetheless, estimates were highly correlated to the latent scores (0.99 for Cognitive Reflection, 0.88 for Calculation) and produced similar regression results.

^2^The conditional probability of answering correctly given that the participant answered non-intuitively cannot be calculated if the participant never answered non-intuitively.

^3^In Study 1, the correlations of Calculation with the latent variable and proportion score were identical if Calculation was scored 0, or if participants who answered all problems intuitively were omitted. This result indicates that this imputation technique is consistent with how the latent variable model treated these participants.

^4^Participants also completed sequences of numbers, but these scores were omitted due to shared variance and construct overlap with numeracy.

^5^Results were nearly identical if Raven's Matrices (as a proxy of fluid intelligence) and vocabulary (as a proxy of crystallized intelligence) were both included as predictors, rather than including the composite, or if all four measures were included separately.

^6^Testing each decision outcome and each financial outcome one at a time while controlling for intelligence was also difficult because excluding participants who did not complete the intelligence measure substantially decreased the sample size for a number of the outcomes. In addition, conducting twelve stepwise regressions would result in excessive Type 1 error rates due to the multiple tests (stepwise regression may result in overfitting, even when conducted just once; Babyak, 2004). Finally, by averaging decision biases, we reduced error. Nonetheless, regressions were also conducted separately for each individual decision bias with similar results that are available from the first author.

^7^Cognitive Reflection was predictive if it was entered without intelligence in the model (*b* = −0.14, *p* = 0.008); however, its effects could still be completely accounted for by numeracy (*b* = −0.03, *p* = 0.60).

^8^ Cognitive Reflection was predictive if it was entered without intelligence in the model (*b* = −0.04, *p* = 0.046); however, its effects could still be completely accounted for by numeracy (*b* = −0.01, *p* = 0.54).

## Appendix

Robust regressions were conducted in order to ensure that the way we chose inattentive or outlier participants did not influence results. The results of these regressions are comparable to those of regular regression, except they are much less susceptible to be influenced by a minority of observations that differ from the general trend. These models are identical to regression models at the first iteration. Observations are iteratively re-weighted according to their distance from the estimated regression line, with further observations being underweighted. The process is repeated until convergence (Holland and Welsch, 1977). Robust regressions were implemented through the MASS package in R (Venables and Ripley, 2002). Below are results reported in Tables 5, 8. Note that demographic variables were also controlled for in this version of Table 5, but are not displayed for simplicity.

**Table A1:** **Robust regression results from Study 1 (see Table 5 for linear regression results)**.

	**Frame Inconsistency**		**Conjunction (subset vs. superset)**		**Conjunction (time)**	
	**Without numeracy**	**With numeracy**	**Without numeracy**	**With numeracy**	**Without numeracy**	**With numeracy**
Intercept	**2.11 (0.20)**	**2.01 (0.20)**	**0.71 (0.10)**	**0.62 (0.10)**	**2.09 (0.22)**	**2.12 (0.22)**
Cognitive Reflection	**−**0.06 (0.05)	**−**0.05 (0.05)	0.03 (0.02)	0.04 (0.02)	0.08 (0.05)	0.07 (0.05)
Calculation	**−0.13 (0.03)**	**−0.10 (0.04)**	**−0.04 (0.02)**	**−**0.02 (0.02)	**−0.08 (0.04)**	**−0.08 (0.04)**
Numeracy	**−**	**−0.11 (0.04)**	**−**	**−0.09 (0.02)**	**−**	0.03 (0.04)
